# Case Report: Cryptococcal Meningitis in a Previously Immunocompetent Patient with Coronavirus Disease 2019

**DOI:** 10.4269/ajtmh.23-0457

**Published:** 2024-01-09

**Authors:** Hyunkyu Kim, Subin Kim, Mi Young Ahn, Dong Hyun Oh, Jae-Phil Choi, Eunmi Yang

**Affiliations:** ^1^ Department of Internal Medicine, Seoul Medical Center, Seoul, South Korea;; ^2^ Division of Infectious Disease, Seoul Medical Center, Seoul, South Korea

## Abstract

*Cryptococcus neoformans* infections occur most frequently in immunocompromised patients. Here, we report a case of cryptococcal meningitis in a previously immunocompetent 78-year-old female patient after treatment of COVID-19. Underlying diseases included hypertension, hyperlipidemia, and diabetes. The patient was critically ill and was treated with remdesivir, baricitinib, and dexamethasone. During hospitalization, her mental state changed, and *C. neoformans* was detected in the cerebrospinal fluid. She died despite receiving antifungal treatment. Treatment of COVID-19 may be a predisposing factor for *C. neoformans* infection. There is a need for concern and countermeasures for opportunistic fungal infections that may accompany COVID-19.

## INTRODUCTION

*Cryptococcus neoformans* infection is an opportunistic fungal infection that occurs mainly in immunocompromised patients, such as those with acquired immune deficiency syndrome, undergoing organ transplant, or who are treated with immunosuppressants or chemotherapy.^1^^,^^2^ In the pandemic caused by SARS-CoV-2, there have been reports of fungal infections associated with COVID-19. To date, the reported cases have mainly been invasive pulmonary aspergillosis, mucormycosis, *Pneumocystis jiroveci*, and candidiasis.^3^^–^^5^ Cryptococcal infections associated with COVID-19 have rarely been reported in immunocompetent patients.^6^^,^^7^ Here, we report a case of cryptococcal meningitis in an immunocompetent patient previously treated with immunosuppressive therapy for COVID-19 in South Korea.

## CASE DESCRIPTION

**Figure 1.**
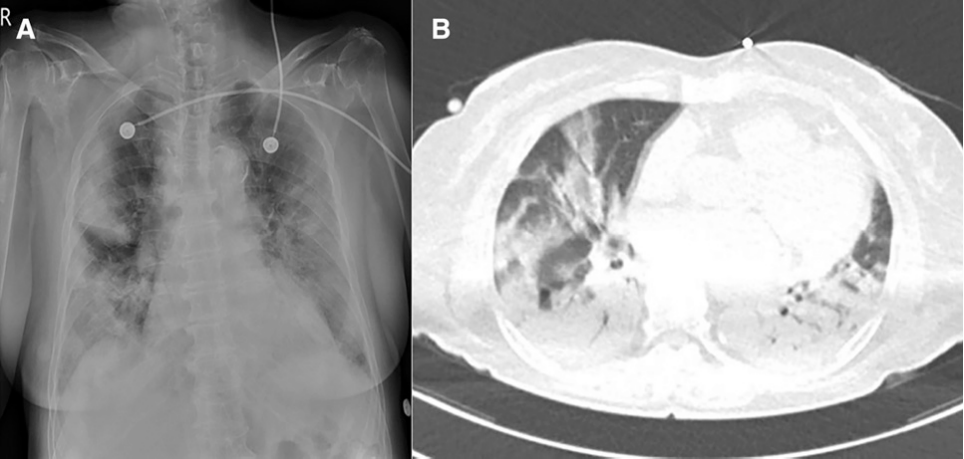
Chest radiograph. (**A**) Bilateral peripheral and lower lung zone opacities of atypical pneumonia. Chest computed tomography. (**B**) Multiple ground-glass opacities in the periphery and lower lobes, consistent with atypical pneumonia with diffuse alveolar damage.

**Figure 2.**
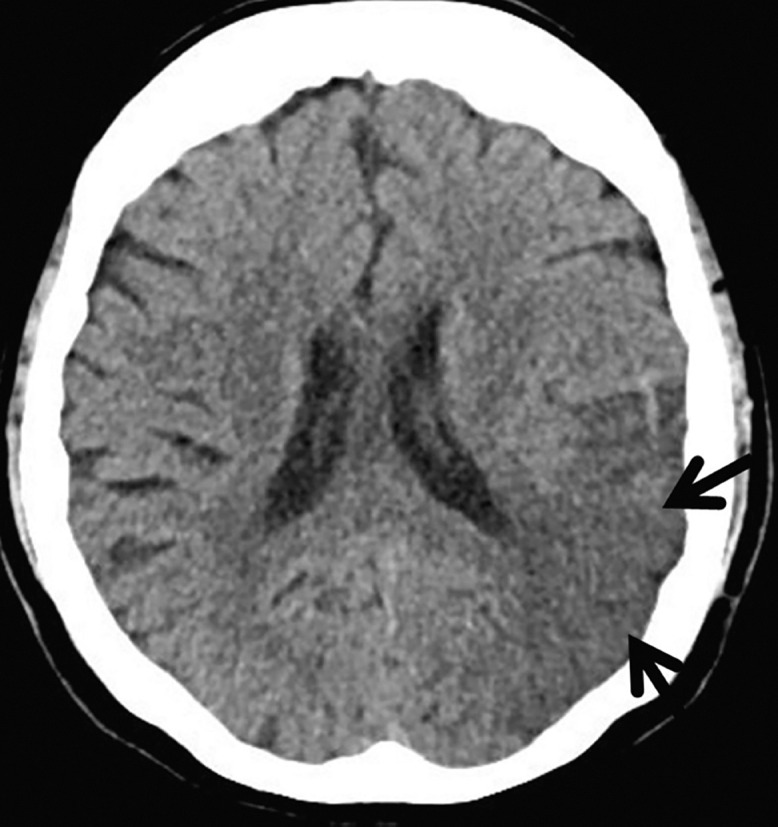
Brain computed tomography shows hypoattenuation in the left middle cerebral artery division, suggesting acute infarction.

A 78-year-old female presented to the emergency department with dyspnea and drowsiness. The underlying diseases were hypertension, hyperlipidemia, and well-controlled diabetes, and she was diagnosed with COVID-19 4 days earlier. The patient was not vaccinated against COVID-19. On physical examination, the mental status was drowsy with severe hypoxia that required supplemental oxygen. Chest radiography showed peripheral and lower zone opacities, and computed tomography (CT) revealed multiple ground-glass infiltrates suggestive of COVID-19 pneumonia (Figure 1). Brain CT showed hypoattenuation of the left middle cerebral artery region, suggesting acute infarction (Figure 2). She was started on mechanical ventilation. Aspirin and clopidogrel were administered to treat brain infarction. Intravenous (IV) piperacillin/tazobactam was administered because combined bacterial pneumonia could not be ruled out. The treatment of COVID-19 pneumonia was initiated with IV remdesivir. Baricitinib (by mouth) 4 mg was administered for 2 weeks as initial treatment combined with IV dexamethasone. Dexamethasone 6 mg was initially administered, followed by 12 mg for 10 days, but her chest radiography findings and O_2_ requirements did not improve; therefore, IV methylprednisolone 1 mg/kg was subsequently administered and tapered for 2 months. The patient underwent a tracheostomy and went to the general ward on day 60 with a mildly drowsy mental state.

**Figure 3.**
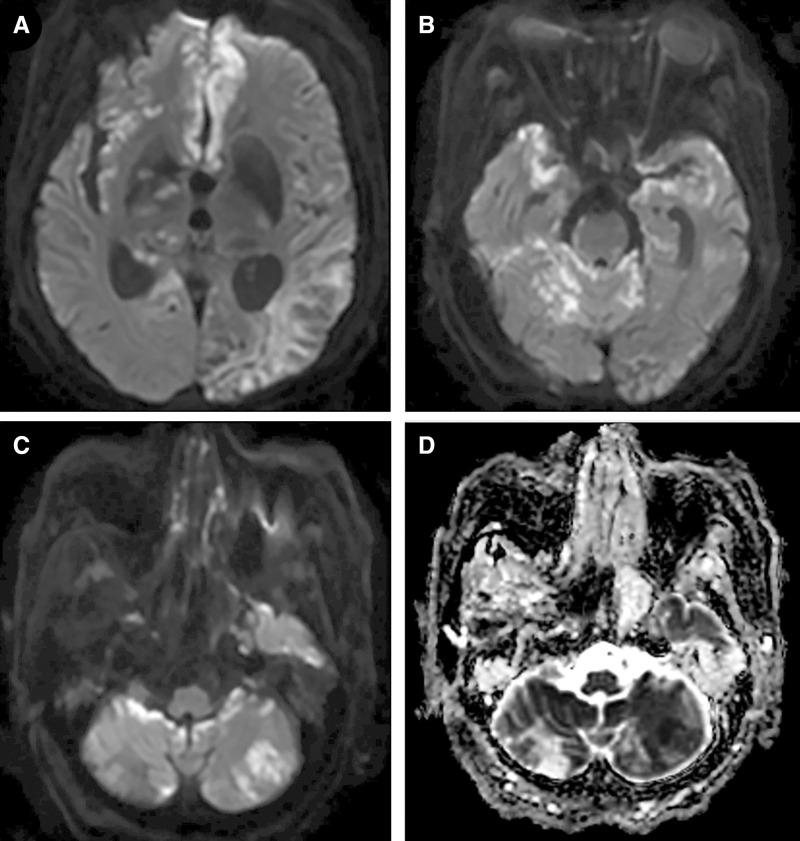
Brain diffusion magnetic resonance imaging. Axial diffusion-weighted image (DWI). (**A**) Hyperintensities in multiple lesions indicative of multifocal acute infarctions. High signal intensity along the right pons and in both cerebellum on axial DWI (**B** and **C**), along with slightly low signal intensity on the axial apparent diffusion coefficient map (**D**), suggest the possibility of meningitis.

On day 86, the patient’s mental status changed from drowsiness to stupor. Brain magnetic resonance imaging revealed changes suggestive of multifocal acute infarctions and meningitis (Figure 3). She underwent spinal tapping, and cerebrospinal fluid analysis revealed a white blood cell count of 140 cells/µL, protein level of 453.7 mg/dL, glucose level of 64 mg/dL, and adenosine deaminase of 24.8 IU/L. The cerebrospinal fluid (CSF) cryptococcal Ag titer was 1:256, and *C. neoformans* was grown in the CSF culture. Induction treatment with liposomal amphotericin B and flucytosine was initiated. After 10 days of treatment, the culture of *C. neoformans* in the CSF was negative. Induction treatment was performed for 3 weeks, followed by consolidation with fluconazole. Despite aggressive management, her state of consciousness did not improve, and she died of septic shock on day 123.

## DISCUSSION

This case is meaningful in that a rare case of cryptococcal meningitis occurred after COVID-19 infection in a patient who previously immunocompetent. Treatment of COVID-19 with corticosteroids and immunomodulatory agents may be a predisposing factor for *C. neoformans* infections. COVID-19 remains an important disease worldwide, and countermeasures are required against opportunistic fungal infections that may accompany this disease.

Cryptococcosis is a fungal infection caused by *Cryptococcus* species, primarily *C. neoformans*. *Cryptococcus* species are ubiquitous in the environment and usually cause invasive infections in immunocompromised individuals. Recently, cryptococcal infections have been reported in patients with COVID-19. Table 1 lists the cases of cryptococcal infection in patients with COVID-19. Cryptococcosis after the diagnosis of COVID-19 occurred more frequently in patients with underlying comorbidities or immunodeficiency, of whom 32% were HIV-infected and 28% were transplant recipients. However, some reports have shown cryptococcosis in patients who did not have predisposing factors at baseline.^6^^–^^8^

**Table 1:** Cases of cryptococcal infection in patients with COVID-19

Case	Age	Sex	Underlying Disease	Treatment of COVID-19	Site of infection/diagnosis	Pathogen	Treatment of cryptococcal infection	Outcome
Choi *et al*.^6^	46	M	None	None	Pulmonary/BAL culture	*C. neoformans*	Fluconazole	Alive
Ghanem *et al*.^7^	73	F	None	Corticosteroid	Meningeal/CSF culture	*C. neoformans*	Amphotericin B + flucytosine	Alive
Thota *et al*.^8^	76	F	HTN, osteoarthritis	Corticosteroid Tocilizumab Convalescent plasma	Disseminated/blood culture, CSF culture	*C. neoformans*	Amphotericin B +	Alive
Gil *et al*.^12^	59	M	HTN, DM, obesity	Corticosteroid	Disseminated/blood culture	*C. neoformans*	Liposomal amphotericin B, fluconazole	Alive
Sharma *et al*.^13^	60	M	HTN, DM, hypothyroidism	Corticosteroid	Pulmonary/lung biopsy	*C. neoformans*	Liposomal amphotericin B, fluconazole	Alive
Alegre-Gonzalez *et al*.^14^	78	M	HTN, DM, CKD	Corticosteroid	Disseminated/blood culture, CSF culture	*C. neoformans*	Amphotericin B + flucytosine, fluconazole	Dead
Karnik *et al*.^15^	57	M	HTN	Corticosteroid	Disseminated/blood culture, CSF culture	*C. neoformans*	Liposomal amphotericin B + flucytosine	Dead
Khatib *et al*.^16^	60	M	HTN, DM, CAD	Corticosteroid Tocilizumab	Disseminated/blood culture	*C. neoformans*	Amphotericin B + flucytosine	Dead
Thyagarajan *et al*.^17^	75	M	HTN, DM, obesity	Corticosteroid Convalescent plasma	Disseminated/blood culture	*C. neoformans*	None	Dead
Traver *et al*.^18^	59	M	COPD, CHF, DM	Corticosteroid Cyclophosphamide	Pulmonary/BAL culture	*C. neoformans*	Liposomal amphotericin B + flucytosine	Dead

COVID-19, coronavirus disease 2019; BAL, bronchoalveolar lavage; CSF, cerebrospinal fluid; HTN, hypertension; DM, diabetes mellitus; CKD, chronic kidney disease; CAD, coronary artery disease; COPD, chronic obstructive pulmonary disease; CHF, congestive heart failure; M, male; F, female.

The association between COVID-19 and cryptococcosis remains unclear. SARS-CoV-2 infection causes immune dysregulation and may affect the T cell response.^9^^,^^10^ Lymphocytes are an important component of the host’s defense mechanism against cryptococcal infection, and alterations in the immune system after COVID-19 may affect cryptococcal infection.^10^^,^^11^ It is also possible that immune suppression therapy with corticosteroids, tocilizumab, and baricitinib may suppress the immune system, resulting in opportunistic infections. Chastain et al.^8^^,^^11^ reported that cryptococcosis with COVID-19 were more likely to have received tocilizumab or baricitinib than those without cryptococcosis. Two reports showed cases of cryptococcosis in patients treated with dexamethasone for COVID-19 without other immune-modulating therapies, and the use of corticosteroids may have contributed to cryptococcosis.^7^^,^^12^

To the best of our knowledge, this is the first case report of cryptococcal meningitis in a patient with COVID-19 in Korea. More cases can be expected during the COVID-19 pandemic, given the widespread use of corticosteroids and immunomodulatory agents to treat COVID-19. We suggest that cryptococcal infection can also occur in immunocompetent hosts with COVID-19 and should be considered an opportunistic infection in patients with COVID-19, especially when treated with immune suppression therapy.
